# Magnetoelectric Vortex Magnetic Field Sensors Based on the Metglas/PZT Laminates

**DOI:** 10.3390/s20102810

**Published:** 2020-05-15

**Authors:** Do Thi Huong Giang, Ho Anh Tam, Vu Thi Ngoc Khanh, Nguyen Trong Vinh, Phung Anh Tuan, Nguyen Van Tuan, Nguyen Thi Ngoc, Nguyen Huu Duc

**Affiliations:** 1 Faculty of Physics Engineering and Nanotechnology, VNU University of Engineering and Technology, Vietnam National University, Hanoi 10000, Vietnam; ngockhanh205vu@gmail.com (V.T.N.K.); trongvinh98@gmail.com (N.T.V.); 2 Laboratory for Micro-Nano Technology, VNU University of Engineering and Technology Vietnam National University, Hanoi 10000, Vietnam; hoanhtam@gmail.com (H.A.T.); ducnh@vnu.edu.vn (N.H.D.); 3 School of Electrical Engineering, Hanoi University of Science and Technology, Hanoi 10000, Vietnam; tuan.phunganh1@hust.edu.vn; 4 Department of Physics, Le Quy Don Technical University, Hanoi 10000, Vietnam; tuannv@lqdtu.edu.vn; 5 Department of Advanced Materials Science and Nanotechnology, University of Science and Technology of Hanoi, Hanoi 10000, Vietnam; nguyen-thi.ngoc@usth.edu.vn

**Keywords:** vortex magnetic sensor, current sensor, magnetoelectric effects, Metglas, closed magnetic circuit

## Abstract

This paper describes the route, from simulations toward experiments, for optimizing the magnetoelectric (ME) geometries for vortex magnetic field sensors. The research is performed on the base of the Metglas/Piezoelectric (PZT) laminates in both open and closed magnetic circuit (OMC and CMC) geometries with different widths (*W*), lengths (*L*), and diameters (*D*). Among these geometries, the CMC laminates demonstrate advantages not only in their magnetic flux distribution, but also in their sensitivity and in their independence of the position of the vortex center. In addition, the ME voltage signal is found to be enhanced by increasing the magnetostrictive volume fraction. Optimal issues are incorporated to realize a CMC-based ME double sandwich current sensor in the ring shape with *D* × *W* = 6 mm × 1.5 mm and four layers of Metglas. At the resonant frequency of 174.4 kHz, this sensor exhibits the record sensitivity of 5.426 V/A as compared to variety of devices such as the CMC ME sensor family, fluxgate, magnetoresistive, and Hall-effect-based devices. It opens a potential to commercialize a new generation of ME-based current and (or) vortex magnetic sensors.

## 1. Introduction

In principle, a multiferroic device has been defined as a combination of two or more primary ferroic ordering phenomena in the same application, such as ferroelectric, ferromagnetic, and ferroelastic. Among its combinations, the ferroelectric–ferroelastic forms the basis of piezoelectric transducers, while the ferromagnetic–ferroelastic is used as piezomagnetic devices. The conventional term “multiferroic” is primarily applied to materials that combine ferroelectricity and ferromagnetism (or in general, magnetism). At present, multiferroics can function with more external stimuli and novel effects, among these, the direct magnetoelectric (ME) effect represents an electric polarization response to an applied magnetic field. This has been employed for technological applications including (AC and (or) DC) magnetic field sensors, transducers, filters, oscillators, phase shifters, transformers or gyrators for voltage gain devices, current sensors, other power conversion devices and electric field tunable microwave magnetic strip line devices. The information on these applications can easily be found in recent review articles and monographs [1,2,3,4]. Multiferroic materials were initially used as single-phase compounds, while they are presently extended to include composites, laminates and nano or micro interlayered structures.

ME laminates of simple disk, square, or rectangular geometries are suitable for sensing magnetic fields of a fixed direction only. In practice; however, AC rotating or vortex magnetic fields exist in many circumstances, particularly in straight wires carrying AC or DC currents (*I*) followed by Ampère’s circuital law H=I/2πR. The first attempt at measuring the current based on the ME effect directly was performed by Bichurin et al. [5], while Dong et al. [6,7,8] suggested that they could detect the vortex magnetic field (and/or current *I*) by using a ring-type ME laminate (called as the O-type). Their ideas were realized for a Terfenol-D/PZT ME ring-type laminate with a ME sensitivity as high as 5.5 V/cm.Oe at the frequency, *f*, of 1 kHz. Generally, one can see that for ring-type ME current sensors, despite the fact of their small size, light weight, and their high sensitivity, the number of publications is still rather modest compared with that of the ME fixed direction magnetic field sensors [3]. Moreover, all these sensors adopt rare-earth elements as the magnetostrictive materials such as Terfenol-D and Samfenol, which is one of the biggest challenges in raw magnetic materials due to their potential supply risk [9,10]. Fortunately, there is an alternative solution to overcome this global supply vulnerability that is the case of the NiCoZn-ferrite (NCZF) [11,12,13] and/or Metglas families [14,15,16,17,18]. Ou et al. [19] recently realized a self-biased current sensor based on the SrFe_12_O_19_/FeCuNbSiB/PZT ME composite cantilever, while Bichurin et al. [15] considered both the resonant and non-resonant type of ME current sensors, which exhibit a sensitivity of 0.34 V/A and 0.53 V/A, respectively. However, these are based on the conventional open magnetic circuit (OMC) in rectangular shape (called as the I-type). To the best of our knowledge, research on the Metglas with closed magnetic circuit (CMC)-based ME current sensors is currently available.

In this paper, attempts are mainly focused on the Metglas-based magnetostrictive O-type ME vortex magnetic sensor. In parallel, the corresponding I-type sensor is also considered. Our studies are fully conducted from simulation of design to experimental realization, and revealed the significant advantages of the no-loss-of-power CMC in the O-type in comparison with the OMC in the ME I-type sensor.

## 2. Experimental

### 2.1. ME Laminate Geometries

**Figure 1:**
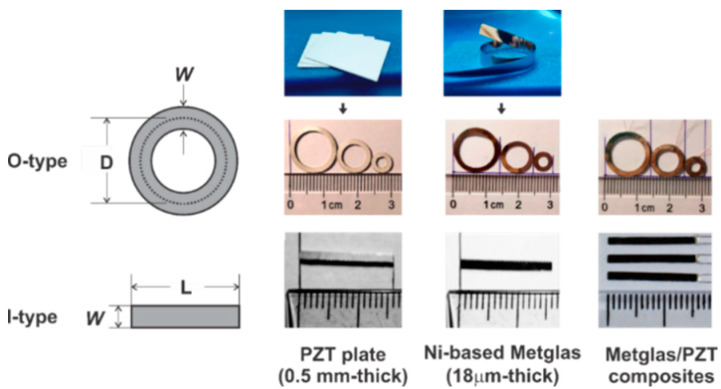
The fabrication processes of ME Metglas/PZT composites of the O-type and I-type.

**Figure 2:**
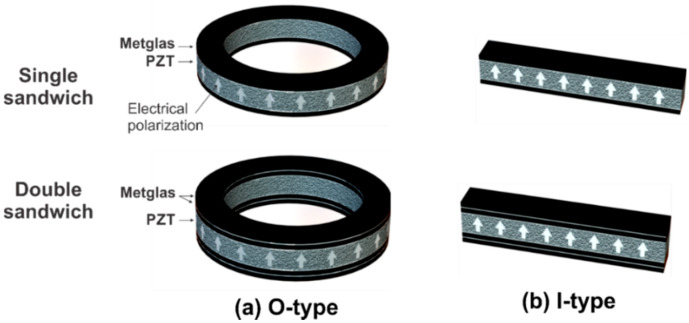
The schematic illustration of different SS (top) and DS (bottom) structures under investigation in the: (**a**) O-type; (**b**) I-type.

Melt-spinning Ni-based Metglas (Met) ribbons with a thickness of 18 μm acting as the magnetic sensitive layers are used due to their high magnetic and magnetostrictive softness. An out-of-plane polarization PZT ceramic plate with 500 μm in thickness (APC 855, APC International, Ltd., Mackeyville, PA, USA) [20] was used for strain mediated electric polarization. By using CNC technology (Bungard CCD/MTC, Windeck, Germany), it was possible to precisely carve both Metglas and PZT segments in ring and rectangular shapes with different sizes (Figure 1). The samples of the ring type are of the same wall width of *W* = 1.5 mm and have various average diameters *D* ranging from 6 mm to 22 mm. A typical rectangular sample of *W* = 1.5 mm in width and *L* = 16 mm in length is likewise collected. The ME composites are prepared by bonding one (single sandwich—SS) or two (double sandwich—DS) magnetic Metglas layers on both at the top and bottom of the PZT plate. A detail about the schematic illustration of different SS and DS structures under investigation is graphically demonstrated in Figure 2. 

### 2.2. ME Effect Characterization Setup

**Figure 3:**
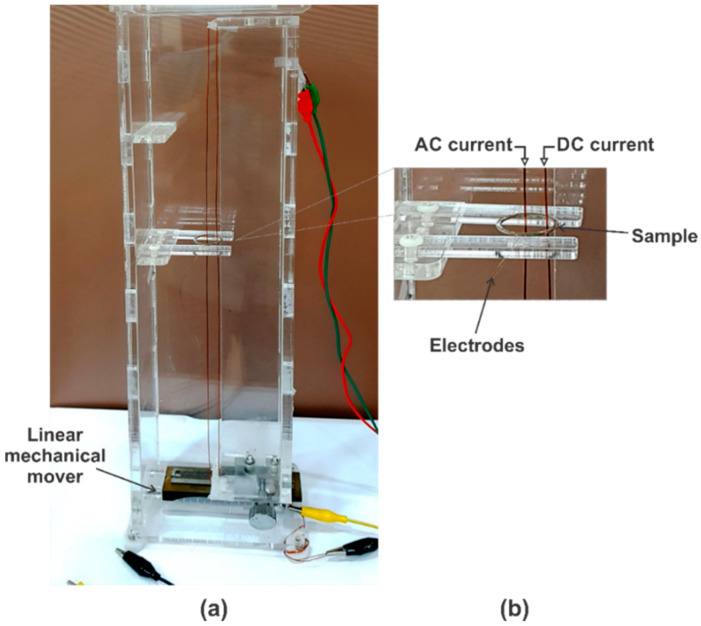
(**a**) The experimental setup for investigation of the dependence of the ME effect on the vortex magnetic field strength and the center displacement; (**b**) The enlarged image at the sample position.

Figure 3 vividly demonstrates a mechanical system for investigating the dependence of the ME effect in the fabricated samples on the vortex magnetic field strength and the center displacement. In the setup, the two straight electrical conductors/wires are placed inside the ME ring. Among these wires, one of which (with an AC current of 0.385 A supplied by the Lock-in amplifier 7265 (Ametek Scientific Instruments, Berwyn, PA, USA) to generate an AC magnetic field for the ME composite operation) is fixed at the ring center, while the vortex magnetic field is created by a DC current from the other wire supplied by the other 2400 Keithley source (Keithley Instruments, Cleveland, OH, USA). The position of the vortex center (or the DC current-carrying wire) can be precisely adjusted by using a linear mechanical mover (Rack and Pinion stage, Edmund Optics Inc., Barrington, NJ, USA). The ME voltage signal is finally measured by the same Lock-in amplifier. 

## 3. Results and Discussion

### 3.1. ME Geometrics Simulation Design

Considerable efforts have been undertaken to elaborate on a phenomenological description of the magnetoelectric voltage coefficient (MEVC), $\alpha_{\mathrm{ME}}=dE/dH$. Although results are still diverse in detail, the MEVC can be generally expressed on the basis of the product of the piezomagnetic and piezoelectric coefficients as follows:

(1) $$\alpha_{\mathrm{ME}}=\frac{dE}{dH}=\frac{\partial E}{\partial \lambda }\frac{\partial \lambda }{\partial H}.$$

In this formula, *λ* represents the magnetostriction of the ferromagnetic phase and ∂λ/∂H is the so-called piezomagnetic coefficient (or magnetostrictive susceptibility *χ*_λ_) of the material; $\frac{\partial E}{\partial \lambda }=\frac{\chi_{p}}{\varepsilon \varepsilon_{ο}}$ is the piezoelectric coefficient (or piezoelectric susceptibility *χ*_p_). Inserting these relations into Equation (1), one obtains:

(2) $$\alpha_{\mathrm{ME}}=\frac{k_{c}\chi_{p}\chi_{\lambda }}{\varepsilon \varepsilon_{ο}},$$

where *k*_c_ is a coupling factor (0 ≤ *k*_c_ ≤ 1), which is of the value between the two (magnetic and electric) phases [3,15]. Thus, the sensor MEVC is directly related to the field dependence of the magnetostriction constant *χ*_λ_. 

Indeed, the (force) magnetostriction is almost quadratically proportional to the magnetization *M* (and thus, the magnetic flux density or magnetic induction *B*) of the magnetic phase, i.e., *λ* ~ *M*^2^) [21]. The ME-based sensor performance and MEVC can be therefore understood partly through the information of the magnetic flux distribution on the Metglas material.

The simulation of the magnetic flux distribution was carefully conducted using the finite element method Ansys Maxwell 3D (Version 16, USA). The measured *B*(*H*) data of Metglas (VSM model 731, Lakeshore Cryotronics, Inc., Westerville, OH, USA) were used as the input parameter in the Magnetostatic mode [22]. In the simulation, the maximum number of elements of 400,000 points and the accuracy of 0.05% were set. The effective magnetic flux taken over the Metglas volume was calculated by:

(3) $$B_{eff}=\frac{1}{V}\int BdV.$$

**Figure 4:**
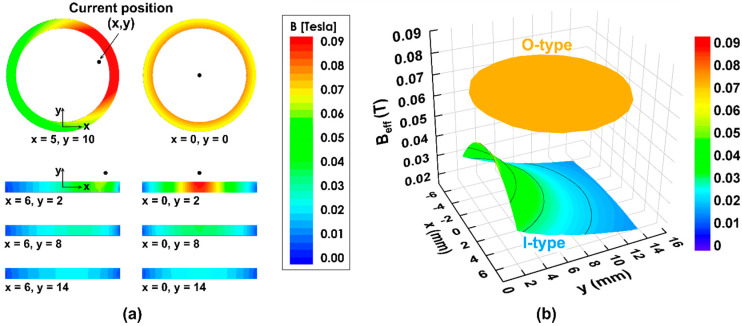
(**a**) The magnetic flux distribution in the vortex magnetic field created by a current of 1A for the 8 mm diameter O-shape (top) and for 1.5 mm × 15 mm I-shape (bottom); (**b**) The corresponding effective magnetic induction *B*_eff_, when the DC electric wire is located at different (*x*,*y*) positions with respect to the sample center.

Figure 4a displays the magnetic flux distribution on the single sandwich SS-Metglas layer in the 16 mm-diameter ring geometry (Figure 4a, top) and the 1.5 mm × 16 mm rectangular one (Figure 4a, bottom). The wire carrying a current of 1 A is located at different (*x*,*y*) positions with respect to the sample center (*x* = 0, *y* = 0). Clearly, the magnetic flux is inhomogeneously distributed over the magnetostrictive layers for both OMC and CMC. However, the effective magnetic flux calculated over the whole sample volume exhibits different behaviors. While the *B*_eff_ value of the I-type is strongly dependent on the location of electric wire, that of the O-type remains almost stable. Actually, the variation of the normalized value of $\left(B_{\mathrm{eff}}^{\max}-B_{\mathrm{eff}}^{\min}\right)/B_{\mathrm{eff}}^{\max}$ is only ~0.7% for the O-type (see the circle plate in Figure 4b). For the rectangular bar; however, this difference varies from 16.6% to 67.7% depending on the position of electric wire close to or far from the center along the *x-* and *y*-directions, respectively (see the saddle horse-type bending in Figure 4b). In addition, *B*_eff_ in the ring shape is about 1.5 times higher than the maximum value in the rectangular one. With regard to the current sensor designs, these issues indicate the advantages of the O-type not only for the sensitivity but also for the position independence.

**Figure 5:**
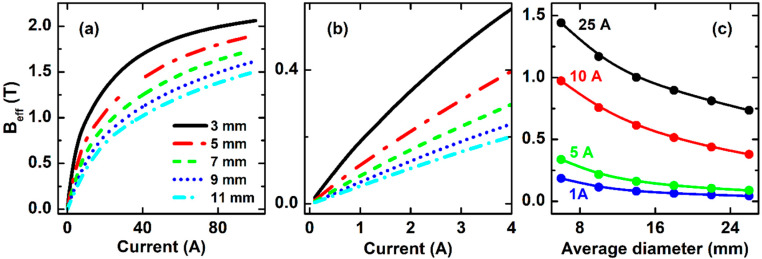
The effective magnetic induction *B*_eff_ data simulated for different current range: (**a**) full range of 0–100 A; (**b**) Low current range; (**c**) Average diameter dependence of *B*_eff_.

The results from simulation of O-types with different diameters are compared in Figure 5. It can clearly be seen that *B*_eff_ decreases with increasing *D*, as expected (Figure 5a,c). However, it is interesting to emphasize that as the ring diameter goes beyond the part of the linear magnetic field dependence of the effective magnetic induction *B*_eff_ is extended further to higher applied currents. For example, in *D* = 7 mm structure, *B*_eff_ decreases one-half as compared with the *D* = 3 mm one, but its linear response range extends to current values up to 4 A (Figure 5b). This is an important design factor to determine the sensor working range. 

**Figure 6:**
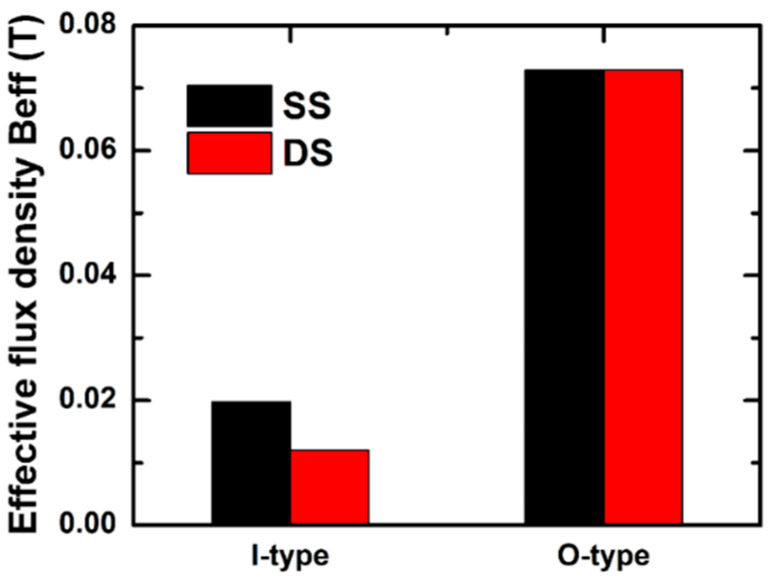
The simulation of the effective magnetic flux density for the SS and DS structures with different numbers of Metglas layers (*n* = 2 and 4, respectively).

In a simple thought, the magnetostrictive strain and consequently the ME effect could be improved by increasing the magnetostriction/PZT volume fraction. To clarify this idea from the point of view of the magnetic flux, the simulation is performed for the SS and DS structures consisting of 2 and 4 Metglas layers, respectively. This expectation, however, does not hold here. In comparison with the SS structure, the *B*_eff_ value obtained in the DS structure is down to 61% in the I-type, whereas it remains almost the same in the O-type for both structures (Figure 6). The lowered *B*_eff_ observed for the I-type may be attributed to the demagnetizing field of adjacent magnetic layers.

### 3.2. Experimental Implementation

**Figure 7:**
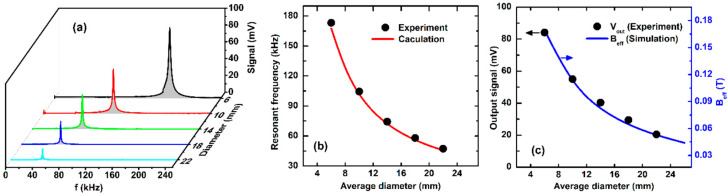
(**a**) The frequency dependence of the ME voltage signal; (**b**) The average diameter dependence of the resonant frequency; (**c**) The ME voltage signal measured for the SS O-type samples of different average diameters.

The dependence of the ME voltage signal on the AC magnetic-field frequency measured at a fixed DC current of 1 A is presented in Figure 7a for the investigated SS ME samples of different diameters, in whichthe resonance behavior is well observed. In addition, the results show that with increasing diameter, the resonance is shifted towards lower frequencies (*f*_r_), whereas the corresponding ME voltage signal significantly decreases. The reduction of the resonance signal seems to agree with the decrease of *B*_eff_ in the Meglas laminates as already mentioned in Figure 5c (see also Figure 7b). The variation of the observed resonant frequency (*f*_r_) can be described by the radiant resonant mode [23]:

(4) $$f_{r}=\frac{1}{\pi D}\sqrt{\frac{1}{\rho s_{11}}},$$

where *ρ* is the average mass density calculated from Metglas and PZT and *S*_11_ is the equivalent elastic compliance. Both quantities are calculated from the mass density, elastic constant and volume fraction of Metglas (*ρ*_m_, $s_{11}^{m}$, *v*_m_) and PZT (*ρ*_p_, $s_{11}^{p}$, *v*_p_) by the following equations [24,25]:

(5) $$\left\{ \begin{array}{l} s_{11}=\frac{v_{m}s_{11}^{m}+v_{p}s_{11}^{p}}{v_{m}+v_{p}}\\ \rho =\frac{v_{m}\rho_{m}+v_{p}\rho_{p}}{v_{m}+v_{p}} \end{array}\right.$$

The experimental data are well fitted (see Figure 7b) with the following parameters for Metglas and PZT layers [20,26]: *ρ*_p_ = 7600 kg/m^3^, *ρ*_m_ = 7180 kg/m^3^, $s_{11}^{p}$ = 16.95 × 10^−12^ m^2^/N, $s_{11}^{m}$ = 9.1 × 10^−12^ m^2^/N. This investigation infers that the resonant frequency can be controlled by changing the ring diameter.

**Figure 8:**
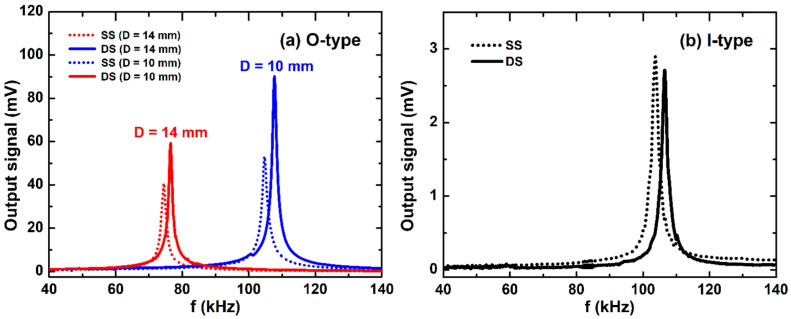
The frequency dependence of the ME voltage signal: (**a**) SS and DS O-type; (**b**) and I-type.

For the DS ME O-type laminates, as illustrated in Figure 8a, the resonance frequency is about 5% lower than the SS ones of the corresponding diameters. The resonant voltage signal, however, is strongly enhanced. Indeed, going from SS to DS, the voltage response is nearly doubled (from 53.09 to 90.18 mV and 31.64 to 59.27 mV) for the samples of *D* = 10 and 14 mm, respectively (see in Table 1). In this case, it seems to agree with the contribution of the enhanced magnetostrictive volume fraction. As discussed in Figure 6, for the DS O-type, the effective magnetic induction B_eff_ in the Metglas layer is almost similar to that of the SS one. Here, the unique difference is that the Metglas/PZT volume fraction is twice enhanced, which leads to the observed enhancement of the resonant signal. This argument is also supported by the experimental data performed for the ME rectangular forms. 

Practically, the frequency dependence of the ME response is presented in Figure 8b for the SS and DS I-type ME laminites with L × W = 15 mm × 1.5 mm. Clearly, there is a rather small modification between these two resonant lines: the resonant frequency and the signal at resonance are slightly shifted from 103 kHz to 108 kHz and from 2.45 mV to 2.24 mV for SS and DS structures, respectively (see also Table 1). For rectangular ME laminates, the resonant frequency was reported to be dependent on the sample length only (*f*_10_ = *v/2L*) [16]. The stability of the observed resonant voltage can be attributed to the combination of the decrease of *B*_eff_ (Figure 6) and the increase of the magnetostrictive volume fraction in the DS sample. Thus, the simulation of the magnetic flux density is useful to comprehend the experimental results and offers helpful information to design CMC for enhanced sensitive current sensors. 

**Figure 9:**
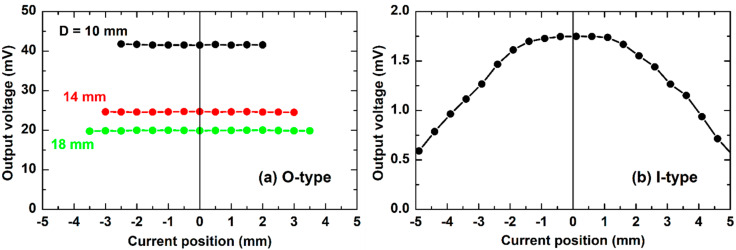
The effect of the vortex center position on the ME output voltage measured for SS samples: (**a**) O-type; (**b**) I-type.

The investigation of the effect of the relative position of the vortex center on the output voltage ME is also carried out. As can be seen from Figure 9, the experimental investigation confirms appropriately the simulation shown in Figure 2c. For the SS O-type (Figure 9a), the signal is perfectly independent on the vortex center position (with an error less than 1%). This error is comparable with that of the integration of six sensors array developed by A. Itzke et al. [27] and Z. Li et al. [28]. For the SS I-type sample (Figure 9b), a huge deviation by about 300% is obtained when moving the vortex center 5 mm along the sample length direction. 

### 3.3. Current Sensor Realization

**Figure 10:**
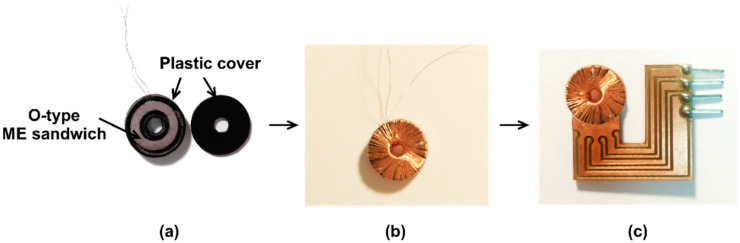
The sensor manufacturing processes: (**a**) packaging in a protective plastic cover; (**b**) winding excitation coil around the case; (**c**) mounting on a PCB board

As regards the high sensitivity, sensors are realized with 6 mm diameter for the SS and DS O-types. The fabrication process is illustrated in Figure 10 for the SS one. The ME ring, after laminating, was packaged in a protective plastic cover (Figure 10a) and the coil was later wrapped around for generating the AC excited magnetic fields (Figure 10b). The sensor was mounted on a PCB for testing (Figure 10c). 

**Figure 11:**
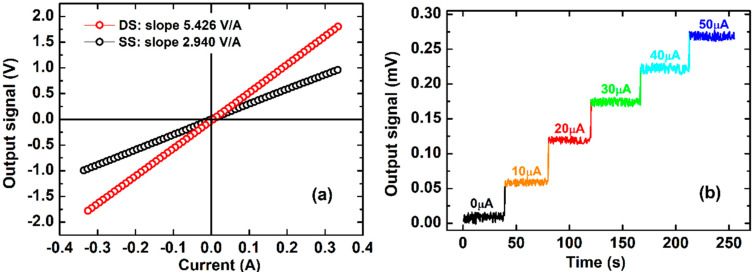
(**a**) The V-I characteristics of the fabricated SS and DS O-type ME-based sensors; (**b**) the output voltage signal of DS O-type ME-based sensors responses to an extremely weak step-varying current of 10 μA.

In the resonant mode, the obtained V-I characteristics of the fabricated sensor are presented in Figure 11a for the SS and DS O-type ME-based sensors. Due to the limitation of the Lock-in amplifier 7265, the investigation is limited up to the output voltage of 0.375 V. The output voltage signal of DS O-type ME-based sensors responses to an extremely weak step-varying current of 10 μA is illustrated in Figure 11b. As can be seen from Figure 11a, the obtained sensor signal exhibits an almost linear behavior in the investigated current range. Sensitivities as high as 2.940 V/A and 5.426 V/A are achieved for the sensors based on the SS and DS O-types, respectively. In fact, the effect of the enhanced magnetostrictive volume fraction in improving the ME sensor sensitivity is worked out. This sensitivity is several tens of times higher than the present record of the ME ring-shape-based current sensors reported for a Terfenol/PZT laminate [29] and for a PZT/NiCoZnO-ferrite trilayer disk [11]. In particular, it is three orders higher than the value of 2.38 mV/A obtained from the ME current sensor using a Terfenol-D/PZT laminate disk inserted into the air gap of a C-shaped ferrite magnetic concentrator [30]. In addition, this sensitivity is one and two orders higher compared to the fluxgate-based sensors [31] and to the sensors based on magnetoresistance [32,33] and Hall effect [34], respectively. The achievable resolution of microampe is several orders of magnitude finer than that of commercial integrated fluxgate current sensors [35]. High sensitivity, low positional dependence error, high temperature stability at room temperature [36,37,38], simple and low-cost production make this CMC ME composite a promising candidate in practical current sensor applications.

This signal processing is performed using the commercial Lock-in amplifier. A current sensor device, however, can be completed thanks to the integration with a home-made digital lock-in amplifier architecture already reported before [39]. The progress will be presented elsewhere.

## 4. Conclusions

A CMC-based current sensor with a record sensitivity of 5.426 V/A with a position-dependent error less than 1% has been designed and manufactured by using magnetic field sensors Metglas/PZT laminates in the ring shape. The innovative achievement is reached thanks to both the optimizing information from the magnetic flux simulation and the enhancement of the magnetostrictive volume fraction. In addition, manufacturing technologies help to realize the mechanical options. With respect to the OMC-based current sensors, the CMC-based sensors demonstrate advantages not only in the sensitivity, but also in the measuring accuracy. It opens a potential to commercialize a new generation of the ME-based current and/or vortex magnetic sensors. 

## Figures and Tables

**Table 1:** The ME voltage signal of the O-type and I-type sandwich structures.

ME Geometries	Dimension (mm)	ME Voltage Signal (mV)	
	(L × W or D × W)	SS	DS
I-type	15 mm × 1.5 mm	2.45	2.45
O-type	10 mm × 1.5 mm	53.09	90.18
	14 mm × 1.5 mm	31.64	59.27
